# Management of ‘floating arm’: a case report of adolescent ipsilateral proximal humerus fracture with open distal complex intraarticular fracture

**DOI:** 10.1093/jscr/rjad724

**Published:** 2024-01-18

**Authors:** Yousef S Alqahtani, Bader N Alotaibi, Lujane S Alqahtani, Ziad A Aljaafri

**Affiliations:** Department of Orthopedic Surgery, Ministry of the National Guard – Health Affairs, Riyadh, Saudi Arabia; King Abdullah International Medical Research Center, Riyadh, Saudi Arabia; Department of Orthopedic Surgery, Ministry of the National Guard – Health Affairs, Riyadh, Saudi Arabia; King Abdullah International Medical Research Center, Riyadh, Saudi Arabia; Department of Orthopedic Surgery, Ministry of the National Guard – Health Affairs, Riyadh, Saudi Arabia; King Abdullah International Medical Research Center, Riyadh, Saudi Arabia; King Abdullah International Medical Research Center, Riyadh, Saudi Arabia; College of Medicine, King Saud bin Abdulaziz University for Health Sciences, Riyadh, Saudi Arabia

**Keywords:** case report, floating arm injury, humerus fracture, MVA, trauma

## Abstract

Simultaneous ipsilateral fractures of the proximal and distal humerus, known as ‘floating arm’, are rarely seen in adolescents and are considered challenging to manage. Most of the published cases have involved proximal humerus and distal supracondylar fractures. This paper presents a special case of floating arm injury in a 14-year-old boy following a motor vehicle accident that was managed in a well-established trauma center. The injury consisted of displaced proximal humerus and open distal T-condylar intraarticular fractures. The patient was discharged in good condition and regained functionality with no reported complications.

## Introduction

Simultaneous ipsilateral fractures of the proximal and distal humerus among children and adolescents are rare. Isolated proximal humerus fractures are the most frequently encountered, following a fall on the outstretched arm [1]. Distal humerus fractures are also common, especially when there is supracondylar involvement and in younger age groups [2]. Conversely, a combination of both fractures leads to a ‘floating arm’ type of injury that is rare. Management of such cases of floating arm injuries is challenging and can have some residual complications [3–5].

A few studies in the literature have presented cases involving proximal humerus and distal supracondylar fracture. This paper presents a special case of floating arm injury that has not previously been described, comprising a displaced proximal humerus ‘surgical neck’ and open distal comminuted T-condylar intraarticular fractures following a motor vehicle accident (MVA) in an adolescent. This was managed operatively with good outcomes.

## Case report

**Figure 1:**
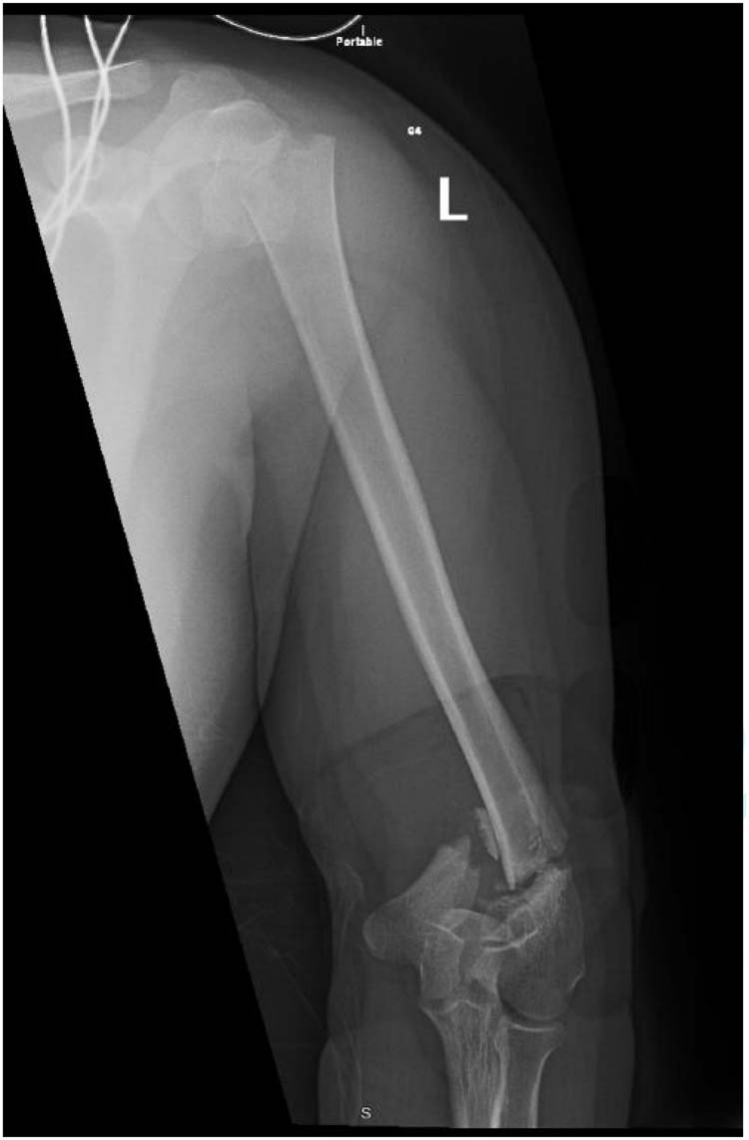
Anteroposterior (AP) view of the left humerus and elbow pre-operative.

A 14-year-old boy with unknown medical and surgical history was brought by the emergency medical services to the emergency department as a case of multiple trauma after a MVA. Upon arrival, primary and secondary surveys were conducted, and management followed accordingly. After stabilization, all related specialties were involved in case management. Multiple fractures were detected, including left mandible, acetabular, open femur, and humerus fractures, as well as a comminuted pelvic fracture. The humerus fractures involved the proximal and distal area, as shown in Fig. 1.

**Figure 2:**
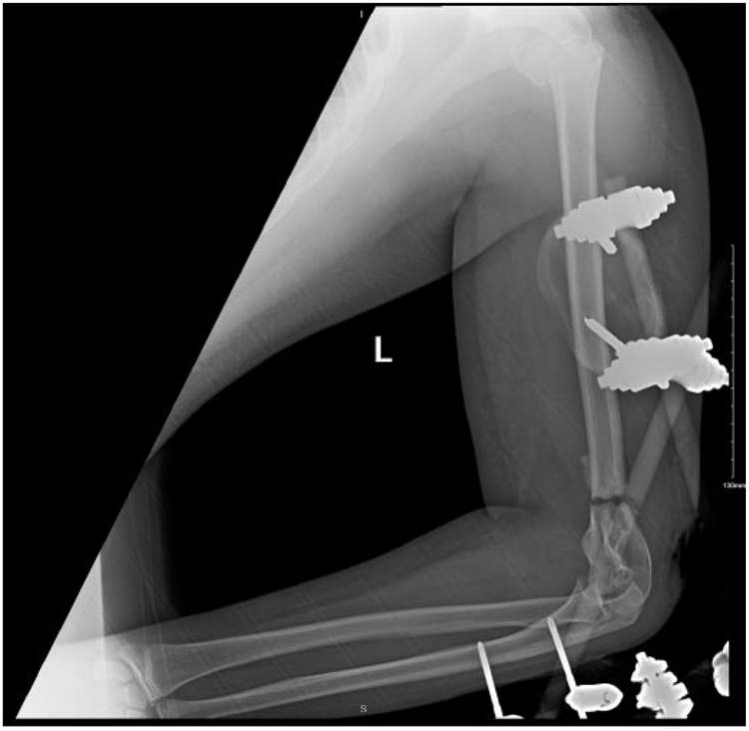
Anteroposterior (AP) view of the left humerus and elbow after Ex-fix application.

**Figure 3:**
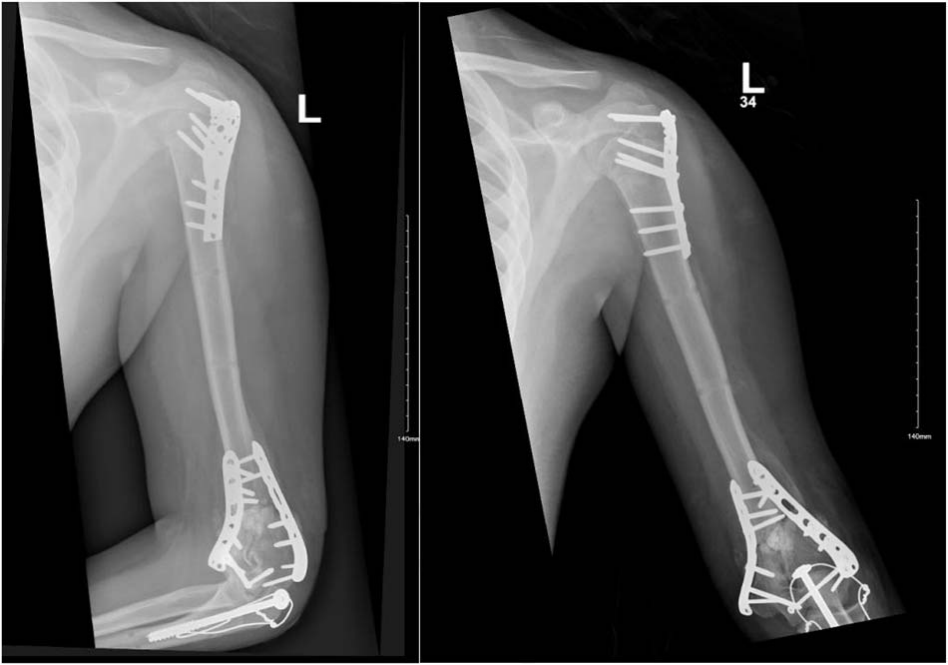
Anteroposterior (AP) view of the left humerus and elbow after ORIF.

Upon arrival the patient underwent exploratory laparotomy followed by irrigation and debridement of both femur and humerus and application of external fixators (Fig. 2). The patient was admitted to the intensive care unit (ICU). Two days later, the patient underwent open reduction and internal fixation of both proximal and distal humerus (Fig. 3).

The patient underwent left open reduction and internal fixation of proximal and distal humerus under general anesthesia. The patient was placed in the lateral position. Preparation and draping in the usual sterile manner were performed and a sterile tourniquet was used. The distal humerus was first approached through a posterior approach. Ulnar nerve protection and olecranon osteotomy was done, showing simple distal intraarticular fracture with minimal metaphyseal comminution. Anatomical reduction of the articular fracture was achieved, with preliminary fixation by k-wires holding the intra-articular fragments. This was followed by interfragmentary screw fixation from medial to lateral. Dual plate fixation (posterolateral and medial anatomical plates) was used to fix both medial and lateral columns, connecting the articular fragment to the metaphyseal. Reduction was confirmed under x-ray. Joint was tested for any screw penetration, and none was found. The osteotomized olecranon was reduced and fixed with cannulated screws and tension bands. The ulnar nerve was protected throughout the whole procedure and was reduced back into the cubital tunnel. Range of motion was examined; full range of motion was achieved. Finally, closure was done in layers.

The patient was then shifted from lateral to beach-chair position, and preparation and draping were repeated. A deltopectoral approach was utilized reaching to the proximal humerus. Reduction was achieved, followed by preliminary fixation with k-wires. Philos plates were used to fix the fracture. Range of motion was examined afterward, with no struggles detected. Fluoroscopic images were taken later to make sure no screws had penetrated the joint. Closure in layers was done, followed by dressing and application of an arm sling.

**Figure 4:**
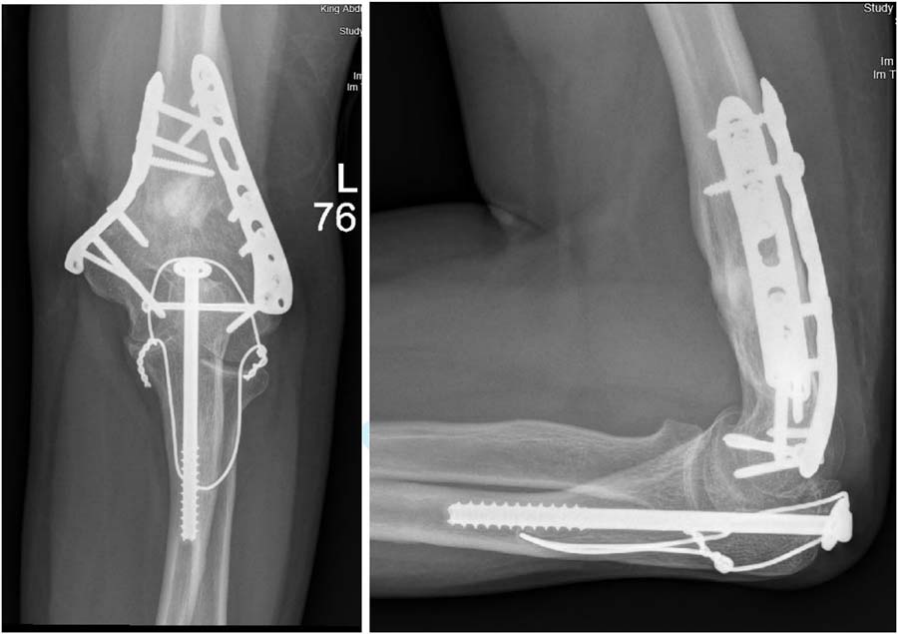
(A). Late follow-up anteroposterior (AP) view of the left elbow after ORIF. (B) Late follow-up lateral view of the left elbow after ORIF.

**Figure 5:**
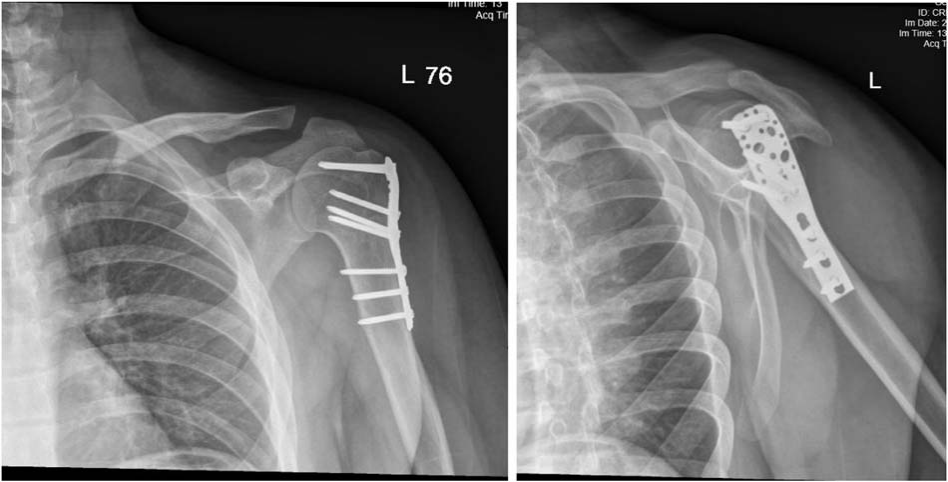
(A) Late follow-up anteroposterior (AP) view of the left shoulder after ORIF. (B) Late follow-up lateral scapula (Y- view) view of the left shoulder after ORIF.

**Figure 6:**
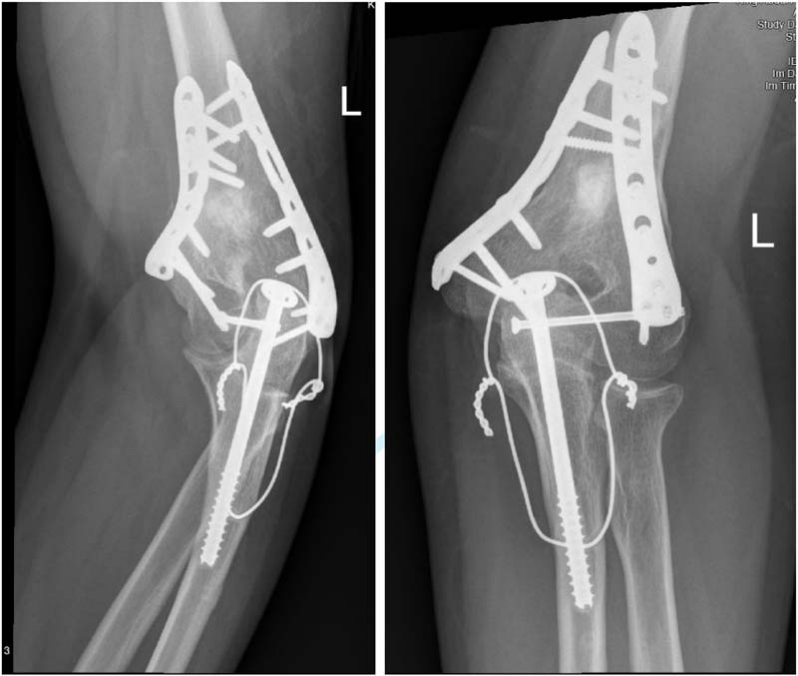
(A) Late follow-up internal oblique view of the left elbow after ORIF. (B) Late follow-up external oblique view of the left elbow after ORIF.

After surgery, the patient was taken back to the ICU and was followed daily by the upper limb orthopedic surgery team for wound care and change of dressings. Four days later, the patient’s condition had improved and he was assessed for distal neurovascular function, which was intact. Afterwards, range of motion was assessed, and no complications were reported before discharge. The patient was kept as an inpatient for almost two more months under the orthopedic trauma team for continuation of management and patient optimization. After discharge, the patient was followed in the orthopedics upper limb and trauma clinic for almost 3 years (Figs 4– 6).

## Discussion

‘Floating arm’ injury, in which simultaneous proximal and distal humerus fractures are encountered, is uncommon. A few articles in the literature have discussed this type of injury and its rarity [3–5].

Management of such a combination of fractures depends on multiple factors that should be kept in consideration. As in previously reported, management of floating arm injury should be initiated by fixing the distal component followed by the proximal [3–5]. Similarly, in our case, we started with the distal fracture. However, olecranon osteotomy was done for better exposure and accurate reduction of the articular surface, followed by application of k-wires and finally plates and locking screws.

A recent retrospective study of distal humerus fracture management showed that it is still debatable whether to give a trial of closed reduction and percutaneous pinning (CRPP) or immediately start with open reduction and internal fixation (ORIF), bearing in mind that choosing CRPP will result in a longer period of immobilization, albeit faster return to a regular range of motion [6]. Previously reported cases [3–5] chose closed reduction and k-wire application, which is feasible in some situations. However, in our patient this was not an option, because of the condition of the soft tissues, a fracture pattern that was intra-articular with significant metaphyseal comminution, and the patient’s multiple site fractures. Olecranon osteotomy is generally performed in situations where distal humerus intraarticular fractures are severely affected: it allows for better exposure for the area hence better reduction [7, 8]. Similarly, in our patient the distal humerus fracture was osteotomized first.

A recent review article studying the outcomes of ORIF versus CRPP for T-condylar humerus fracture reported that most cases were managed with ORIF in order to achieve anatomical congruity, especially for medial and lateral columns, and to stabilize intra-articular fragments [9].

Our patient was followed afterwards for three years with serial x-rays. No signs of growth arrest or deformity due to our surgical intervention were noted. Instead, on follow up, our patient had a functional range of motion. Elbow range of motion was documented to be 30° extension to almost full flexion and intact pronation and supination. The range of shoulder motion was documented as full. Good healing was observed, but there was some hardware prominence that slightly affected the range of motion, and the patient will be booked for hardware removal. The patient was happy and satisfied about the overall outcome and restored functionality.

## Conclusion

Open reduction and internal fixation with olecranon osteotomy to achieve anatomical reduction of articular involvement is feasible and safe in adolescent humeral fractures. No growth impairment or deformity was noted from the olecranon osteotomy, and functionality of the patient was restored.
